# Effect of spectacle lenses with aspherical lenslets on choroidal thickness in myopic children: a 3-year follow-up study

**DOI:** 10.1186/s40662-024-00383-4

**Published:** 2024-04-25

**Authors:** Yingying Huang, Xue Li, Zuopao Zhuo, Jiali Zhang, Tianxing Que, Adeline Yang, Björn Drobe, Hao Chen, Jinhua Bao

**Affiliations:** 1 National Engineering Research Center of Ophthalmology and Optometry, Eye Hospital, Wenzhou Medical University, 270 West Xueyuan Road, Wenzhou, Zhejiang 325027 China; 2 Wenzhou Medical University – Essilor International Research Center (WEIRC), Wenzhou Medical University, Wenzhou, Zhejiang China; 3 School of Biomedical Engineering, Wenzhou Medical University, Wenzhou, Zhejiang China; 4 R&D Singapore, Essilor International, Singapore, Singapore

**Keywords:** Choroidal thickness, Myopia control, Aspherical lenslets, Crossover

## Abstract

**Background:**

To investigate the impact of wearing spectacle lenses with highly aspherical lenslets (HAL) for 3 years and the impact of switching from single-vision lenses (SVL) to HAL on choroidal thickness (ChT).

**Methods:**

Fifty-one participants who had already worn HAL for 2 years continued wearing them for an additional year (HAL group). Further, 50 and 41 participants who had worn spectacle lenses with slightly aspherical lenslets (SAL) and SVL for 2 years, respectively, switched to wearing HAL for another year (SAL-HAL and SVL-HAL groups). Additionally, 48 new participants aged 10–15 years were enrolled to wear SVL at the third year (new-SVL group). ChT was measured every 6 months throughout the study.

**Results:**

Significant differences were observed in the changes in ChT among the four groups at the third year (all *P* < 0.05 except for the outer nasal region: *P* = 0.09), with the new-SVL group showing larger reductions compared with the other three groups. However, none of the three HAL-wearing groups showed significant changes in ChT at the third year (all *P* > 0.05). When comparing the changes in ChT for 3 years among the HAL, SAL-HAL, and SVL-HAL groups, significant differences were found before switching to HAL, but these differences were abolished after all participants switched to HAL.

**Conclusions:**

Compared to those in the SVL group, choroid thinning was significantly inhibited in all the HAL groups. Wearing HAL for 3 years no longer had a choroidal thickening effect but could still inhibit choroidal thinning compared to wearing SVL.

**Trial registration:**

The study was registered at the Chinese Clinical Trial Registry (ChiCTR1800017683), http://www.chictr.org.cn/showproj.aspx?proj=29789.

## Background

The choroid is a highly vascular tissue located between the retina and sclera. It supplies oxygen and nutrients to the outer retina and sclera and is hypothesized to play an important role in myopia progression and control. Animal studies have found that one possible mechanism of myopia is scleral hypoxia caused by a decrease in choroidal blood flow [1–3]. Studies have shown a significant reduction in choroidal thickness (ChT) accompanying myopia incidence and progression in children [4, 5]. The use of myopia control interventions i.e., low concentrations of atropine [6], orthokeratology lenses [7], multifocal contact lenses [8], and spectacle lenses with myopic defocus [9] for 1 or 2 years resulted in significant increases in the ChT.

Previously, we found that spectacle lenses with highly aspherical lenslets (HAL, Stellest, Essilor [10, 11]) significantly reduced myopia progression and slowed axial length (AL) elongation over 2 years [12]. Children in the single-vision lens (SVL) group exhibited sustained choroidal thinning over 2 years, while those in the HAL group exhibited significant choroidal thickening [13]. This study was the second stage of the previous study. The control group who originally wore SVL and the other test group who wore spectacle lenses with slightly aspherical lenslets (SAL) transition to wearing HAL for the third and final year of the study, while the participants who originally wore HAL continued to do so [14]. This study aimed to investigate the impact of wearing HAL for 3 years and the impact of switching from the SAL and SVL to the HAL on the choroid.

## Methods

### Study design

Participants who completed the 2-year randomized clinical trial were invited to participate in the extended study for an additional year. Those who had worn HAL for 2 years continued wearing them in the third year (HAL group), while participants from the original SAL or SVL groups switched to wearing HAL in the third year (SAL-HAL group and SVL-HAL group); additionally, a new control group (new-SVL group) was recruited in the third year [14]. The inclusion criteria for the new-SVL group were based on the participants from the SVL-HAL group at the extended baseline (the end of the 2-year visit) i.e., aged between 10 and 15 years with a spherical equivalent refractive error (SER) between −1.75 and −6.00 D. This study was approved by the Ethics Committee of the Eye Hospital of Wenzhou Medical University (Y2018-054-02), and all the work was conducted following the tenets of the Declaration of Helsinki. All the participants were informed about the changes in the study design after the 2-year trial and were then invited to wear HAL in the third year of the study. Written informed consent was obtained again for the extended third year study. For the new-SVL group, ﻿written informed consent was obtained from the subjects and their parents or guardians after providing verbal and written explanations of the objectives and possible consequences of the study.

### Measurements

Two drops of 1% cyclopentolate were administered at 5-min intervals for cycloplegia. Measurements were taken 30 min after the last drop. The SER in the right eye was obtained using an autorefractor (KR-800, TOPCON Corp, Tokyo, Japan). The AL in the right eye was measured with a Lenstar (LS 900, Haag-Streit AG, Koeniz, Switzerland). The average values of ten SER measurements and five AL measurements were used for data analysis.

The ChT was measured in the right eye using a swept-source optical coherence tomography (OCT) device (DRI OCT Triton, Topcon Corporation, Tokyo, Japan). The subfoveal (SF) ChT was measured three times with a 9-mm line scan composed of 128 single B-scans. Twelve-line radial scans with a 6-mm scan length centered on the fovea were obtained three times to determine the average ChT of the different regions. Each radial OCT image was constructed from an average of 16 B-scans and contained 1024 axial scans each. A central circular region measuring 6 × 6 mm was automatically partitioned according to the Early Treatment Diabetic Retinopathy Study (ETDRS). The nine regions were classified as follows: the central fovea, consisting of a 1 × 1 mm circular region (C); and the nasal, superior, temporal and inferior regions measured at 1–3 mm (N3, S3, T3 and I3) and 3–6 mm (N6, S6, T6 and I6). The built-in software automatically provided the thickness in each direction and calculated the average thickness of three images in each area. The central area was the average of all 12 images. The ChT was determined as the thickness between the outer retinal pigment epithelium and the inner choroidoscleral interface. Manual corrections for boundaries at both the retinal-choroidal and choroidal-scleral interfaces were conducted by two masked and well-trained independent observers. Both observers manually corrected all the OCT images for every participant. The images were reanalyzed if the difference between the results from the two observers was greater than 10 µm. The instrument adjusted the scan length and the ChT using Littman formula based on the AL and SER of the participant at each visit [15]. The results from the three measurements obtained by the two observers were averaged and used for data analysis. Intraobserver repeatability analysis of the three repeated images at each visit and interobserver repeatability analysis between the two observers were also evaluated, and the results revealed that both had good consistency (all ICCs > 0.994 in all choroidal regions).

### Statistical analysis

IBM SPSS Statistics v.24.0 was used for the data analysis. Repeated-measures multivariate analysis of variance (RM-MANOVA) was conducted to assess the impact of time and group on the ChT, with each region (nine regions and SF) as a separate variable adjusted for age, sex, baseline SER and AL. MANOVA with adjustments for age, sex, baseline SER and AL was performed to compare the 6-month and 12-month changes in the ChT between groups; post hoc analysis was also conducted using the Bonferroni correction for multiple comparisons. Pearson correlation analysis was used to evaluate the associations between changes in AL and changes in ChT within each group. Linear regression analysis was used to explore the correlation between the progression of AL and baseline ChT as well as age, sex, SER, and AL within each group. MANOVA adjusted for initial (3 years prior) age, sex, SER and AL was used to compare the 3-year changes in the ChT among the HAL, SAL-HAL and SVL-HAL groups. After adjusting for ten variables, *P* < 0.005 was considered to indicate statistical significance in the ChT comparisons, while *P* < 0.05 was considered to indicate statistical significance in other analyses.

## Results

A total of 51 participants in the HAL group, 50 participants in the SAL-HAL group, 41 participants in the SVL-HAL group and 48 participants in the new-SVL group completed the third year of follow-up and all examinations. The baseline demographic and ocular characteristics of the participants in each group are shown in Table 1. There were significant differences in age, SER and AL among the four groups (all *P* ≤ 0.03), but no significant differences were found between the SVL-HAL group and the new-SVL group (all *P* ≥ 0.46). Significant differences were observed in the changes in the AL among the four groups (both *P* < 0.001, Table 1). The HAL, SAL-HAL, and SVL-HAL groups showed smaller increases in the AL than the new-SVL group (all *P* ≤ 0.002), but no significant differences were observed between the HAL, SAL-HAL, and SVL-HAL groups (all *P* ≥ 0.08).

**Table 1:** Baseline characteristics of the participants in each group

Parameter	HAL group	SAL-HAL group	SVL-HAL group	New-SVL control group	ANOVA *P* value	SVL-HAL *vs.* new-SVL *P* value
No. of participants	51	50	41	48	NA	NA
Age (years)	12.67 ± 1.16	12.10 ± 1.25	12.10 ± 1.22	11.98 ± 1.28	0.0025	> 0.999^a^
Sex (M/F)	25/26	16/34	23/18	22/26	0.122	NA
Baseline SER (D)	−3.32 ± 1.11	−3.35 ± 1.04	−3.86 ± 0.88	−3.73 ± 1.00	0.024	> 0.999^a^
Baseline AL (mm)	25.10 ± 0.78	24.92 ± 0.79	25.38 ± 0.64	25.47 ± 0.80	0.001	> 0.999^a^
6-month AL elongation (mm)	0.10 ± 0.08	0.10 ± 0.09	0.08 ± 0.09	0.16 ± 0.07	< 0.001^b^	< 0.001^a^
1-year AL elongation (mm)	0.17 ± 0.12	0.18 ± 0.14	0.15 ± 0.14	0.28 ± 0.11	< 0.001^b^	< 0.001^a^

HAL group, continued wearing HAL; SAL-HAL group, switched from wearing SAL to HAL; SVL-HAL group, switched from wearing SVL to HAL; new-SVL group, wore SVL for 1 year

*P* < 0.05 indicates statistical significance

*HAL* = spectacle lenses with highly aspherical lenslets; *SAL* = spectacle lenses with slightly aspherical lenslets; *SVL* = single vision spectacle lenses; *AL* = axial length; *SER* = spherical equivalent refractive error; *NA* = not applicable

^a^Bonferroni corrected

^b^Adjusted for age, sex, baseline SE and AL

**Fig. 1:**
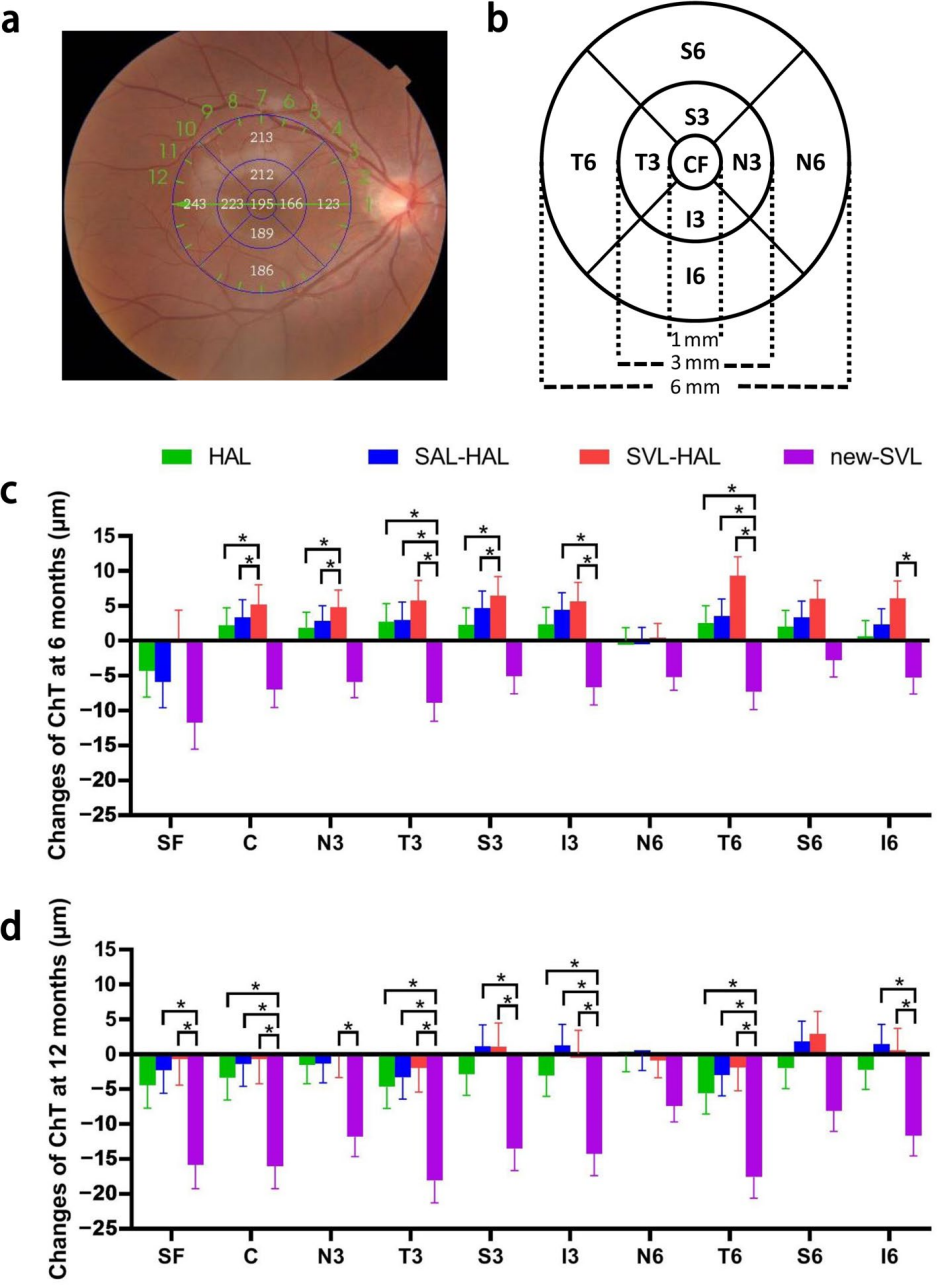
The regions of the choroid are shown in the image (**a**) and schematic diagram (**b**), and 6-month (**c**) and 12-month (**d**) changes in choroidal thickness (ChT) were compared among in the four groups. Changes in ChT were adjusted for age, sex, baseline axial length and refractive error. SF, subfoveal choroidal thickness; C, 1 × 1 mm circular region; N3, S3, T3 and I3, the nasal, superior, temporal and inferior regions measured at 1–3 mm; N6, S6, T6 and I6, the nasal, superior, temporal and inferior regions measured at 3–6 mm; HAL, spectacle lenses with highly aspherical lenslets; SAL, spectacle lenses with slightly aspherical lenslets; SVL, single vision lenses. HAL group, continued wearing HAL; SAL-HAL group, switched from wearing SAL to HAL; SVL-HAL group, switched from wearing SVL to HAL; new-SVL group, wore SVL for 1 year. *P < 0.05 in the post hoc tests conducted using Bonferroni correction

A comparison of the ChT of each region among the four groups by RM-MANOVA revealed that the main effects of time and group were not significant. However, there were significant interactions between time and group in some regions (Table 2). RM-MANOVA within each group revealed a significant reduction in the ChT over time in the new-SVL group (all *P* ≤ 0.005, Table 2). In contrast, no significant changes were observed in the HAL, SAL-HAL or SVL-HAL groups. The changes in the ChT were significantly different among the four groups (Fig. 1c & d, Table 3).

**Table 2:** Choroidal thickness adjusted for age, sex, baseline axial length and refractive error in different regions

**Time**	**Group**	**SF**	**C**	**N3**	**T3**	**S3**	**I3**	**N6**	**T6**	**S6**	**I6**
Baseline	HAL (μm)	236.83 ± 58.03	238.59 ± 57.16	213.64 ± 54.55	252.58 ± 56.47	249.32 ± 55.3	240.37 ± 55.97	170.91 ± 49.18	259.47 ± 52.82	253.47 ± 54.22	234.03 ± 52.58
	SAL-HAL (μm)	227.8 ± 57.71	230.72 ± 56.84	209.6 ± 54.24	245.33 ± 56.15	241.66 ± 54.98	238.96 ± 55.66	171.23 ± 48.9	255.65 ± 52.52	247.32 ± 53.92	241.26 ± 52.28
	SVL-HAL (μm)	215.96 ± 57.4	217.1 ± 56.54	195.81 ± 53.97	231.47 ± 55.86	225.12 ± 54.7	225.18 ± 55.37	159.04 ± 48.65	237.81 ± 52.25	230.83 ± 53.65	219.97 ± 52.01
	new-SVL (μm)	234.2 ± 58.04	233.22 ± 57.17	205.69 ± 54.56	250.6 ± 56.48	242.3 ± 55.31	237.17 ± 55.99	161.49 ± 49.19	258.4 ± 52.83	242.84 ± 54.23	227.4 ± 52.59
6 months	HAL (μm)	232.45 ± 62.94	240.65 ± 59.02	215.55 ± 56.84	255.14 ± 57.97	251.62 ± 57.66	242.76 ± 58.93	171.18 ± 51.8	261.9 ± 53.92	255.66 ± 56.88	234.88 ± 56.2
	SAL-HAL (μm)	221.96 ± 62.59	234.06 ± 58.69	212.45 ± 56.52	248.33 ± 57.65	246.35 ± 57.33	243.41 ± 58.61	171.49 ± 51.51	259.11 ± 53.62	250.65 ± 56.56	243.83 ± 55.88
	SVL-HAL (μm)	216.16 ± 62.26	222.3 ± 58.38	200.29 ± 56.23	237.49 ± 57.35	231.52 ± 57.04	230.55 ± 58.3	159.09 ± 51.24	247.63 ± 53.34	236.78 ± 56.27	225.49 ± 55.6
	new-SVL (μm)	222.4 ± 62.95	226.23 ± 59.04	199.77 ± 56.85	241.65 ± 57.98	237.24 ± 57.67	230.51 ± 58.95	156.14 ± 51.8	250.96 ± 53.93	240.03 ± 56.89	221.95 ± 56.21
12 months	HAL (μm)	231.74 ± 58.69	234.66 ± 58.51	211.85 ± 56.17	247.27 ± 56.37	245.97 ± 56.62	237.13 ± 58.22	170.71 ± 51.6	253.14 ± 51.65	251.22 ± 56.24	231.76 ± 55.41
	SAL-HAL (μm)	225.58 ± 58.36	229.19 ± 58.18	208.11 ± 55.86	241.98 ± 56.06	242.73 ± 56.3	240.05 ± 57.89	171.18 ± 51.31	252.42 ± 51.36	249.02 ± 55.93	242.57 ± 55.1
	SVL-HAL (μm)	215.84 ± 58.05	216.72 ± 57.88	195.5 ± 55.57	230.36 ± 55.77	226.68 ± 56	225.22 ± 57.59	157.91 ± 51.05	237.37 ± 51.09	234.01 ± 55.64	220.38 ± 54.81
	new-SVL (μm)	218.1 ± 58.7	217.11 ± 58.52	193.87 ± 56.18	232.22 ± 56.39	228.73 ± 56.62	222.88 ± 58.22	154.05 ± 51.62	240.44 ± 51.66	234.8 ± 56.25	215.77 ± 55.42
RM-MANOVA, *P* value	Time	0.661	0.372	0.525	0.551	0.515	0.431	0.942	0.396	0.468	0.937
	Group	0.550	0.433	0.429	0.465	0.305	0.573	0.378	0.414	0.321	0.192
	Time × group	0.100	0.006	0.025	0.001	0.005	0.003	0.175	< 0.001	0.086	0.009
RM-MANOVA in each group, *P* value	HAL	0.434	0.116	0.311	0.037	0.115	0.112	0.892	0.014	0.174	0.375
	SAL-HAL	0.384	0.173	0.187	0.066	0.210	0.211	0.964	0.051	0.455	0.583
	SVL-HAL	0.983	0.173	0.166	0.094	0.107	0.147	0.926	0.003	0.119	0.095
	new-SVL	< 0.001	< 0.001	< 0.001	< 0.001	< 0.001	< 0.001	0.001	< 0.001	0.005	< 0.001

HAL group, continued wearing HAL; SAL-HAL group, switched from wearing SAL to HAL; SVL-HAL group, switched from wearing SVL to HAL; new-SVL group, wore SVL for 1 year

*P* < 0.005 indicates statistical significance

*HAL* = spectacle lenses with highly aspherical lenslets; *SAL* = spectacle lenses with slightly aspherical lenslets; *SVL* = single vision spectacle lenses; *SF* = subfoveal choroidal thickness; *C* = 1 × 1 mm circular region; *N3, S3, T3 and I3* = the nasal, superior, temporal and inferior regions measured at 1–3 mm; *N6, S6, T6 and I6* = the nasal, superior, temporal and inferior regions measured at 3–6 mm; *RM-MANOVA* = repeated-measures multivariate analysis of variance

**Table 3:** Changes in choroidal thickness in the third year in the four groups (mean ± SE)

**Time**	**Group**	**SF**	**C**	**N3**	**T3**	**S3**	**I3**	**N6**	**T6**	**S6**	**I6**
Changes at 6 months	HAL (μm)	−4.26 ± 3.70	2.20 ± 2.53	1.90 ± 2.20	2.35 ± 2.44	2.75 ± 2.58	2.37 ± 2.45	0.18 ± 1.83	2.16 ± 2.33	2.62 ± 2.43	0.78 ± 2.26
	SAL-HAL (μm)	−5.87 ± 3.71	3.35 ± 2.54	2.84 ± 2.21	4.7 ± 2.45	2.99 ± 2.58	4.43 ± 2.46	0.16 ± 1.84	3.32 ± 2.33	3.53 ± 2.44	2.33 ± 2.28
	SVL-HAL (μm)	0.01 ± 4.10	4.98 ± 2.81	4.49 ± 2.44	6.32 ± 2.71	5.65 ± 2.86	5.43 ± 2.72	0.18 ± 2.02	6.00 ± 2.58	9.30 ± 2.71	5.79 ± 2.50
	new-SVL (μm)	−11.73 ± 3.81	−6.94 ± 2.61	−5.92 ± 2.27	−5.05 ± 2.51	−8.83 ± 2.65	−6.67 ± 2.53	−5.26 ± 1.88	−2.80 ± 2.40	−7.28 ± 2.51	−5.35 ± 2.33
MANOVA, *P* values		0.205	0.007	0.008	0.001	0.010	0.004	0.109	< 0.001	0.080	0.010
Changes at 12 months	HAL (μm)	−4.53 ± 3.33	−3.48 ± 3.16	−1.54 ± 2.76	−2.86 ± 3.06	−4.73 ± 3.13	−3.09 ± 3.04	−0.18 ± 2.27	−1.93 ± 2.91	−5.69 ± 2.99	−2.22 ± 2.83
	SAL-HAL (μm)	−2.34 ± 3.33	−1.52 ± 3.17	−1.39 ± 2.77	1.15 ± 3.06	−3.38 ± 3.13	1.20 ± 3.05	−0.03 ± 2.28	1.81 ± 2.91	−3.00 ± 3.00	1.50 ± 2.86
	SVL-HAL (μm)	−1.11 ± 3.68	−1.12 ± 3.50	−0.69 ± 3.05	0.74 ± 3.39	−2.28 ± 3.47	−0.33 ± 3.37	−1.15 ± 2.51	2.70 ± 3.22	−2.15 ± 3.32	0.20 ± 3.14
	new-SVL (μm)	−15.73 ± 3.42	−15.96 ± 3.25	−11.85 ± 2.84	−13.47 ± 3.14	−17.97 ± 3.22	−14.24 ± 3.13	−7.45 ± 2.34	−8.08 ± 2.99	−17.43 ± 3.08	−11.69 ± 2.92
MANOVA, *P* values		0.012	0.004	0.017	0.002	0.004	0.003	0.086	0.002	0.050	0.007

HAL group, continued wearing HAL; SAL-HAL group, switched from wearing SAL to HAL; SVL-HAL group, switched from wearing SVL to HAL; new-SVL, wore SVL for 1 year

*P* < 0.005 indicates statistical significance

*HAL* = spectacle lenses with highly aspherical lenslets; *SAL* = spectacle lenses with slightly aspherical lenslets; *SVL* = single vision spectacle lenses; *SF* = subfoveal choroidal thickness; *C* = 1 × 1 mm circular region; *N3, S3, T3 and I3* = the nasal, superior, temporal and inferior regions measured at 1–3 mm; *N6, S6, T6 and I6* = the nasal, superior, temporal and inferior regions measured at 3–6 mm; *MANOVA* = multivariate analysis of variance

The third-year changes in the ChT were significantly negatively correlated with changes in the AL in the HAL group (all *P* ≤ 0.003, *R* ranging from −0.37 to −0.52) and the SAL-HAL group (all *P* < 0.001, *R* ranging from −0.59 to −0.66) across all regions examined. However, no significant correlations were observed in the SVL-HAL or new-SVL groups (all *P* ≥ 0.07). Baseline age and AL were found to be correlated with 1-year AL increases within the HAL group (*P* < 0.001, *r*^2^ = 0.30), while the baseline ChT in region C was correlated with AL increases within the SAL-SVL group (*P* = 0.03, *r*^2^ = 0.09). Changes in the AL within the SVL-HAL and new-SVL groups were not correlated with baseline age, sex, AL, SER, or ChT.

**Fig. 2:**
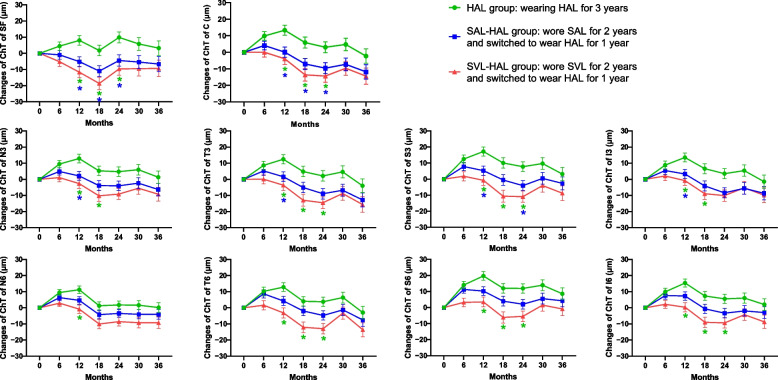
Three-year changes in choroidal thickness (ChT) in the three groups. Changes in ChT were adjusted for age, sex, baseline axial length and refractive error. The blue asterisks represent significant differences between the SAL-HAL and SVL-HAL groups, while the green asterisks represent significant differences between the HAL and SVL-HAL groups. SF, subfoveal choroidal thickness; C, 1 × 1 mm circular region; N3, S3, T3 and I3, the nasal, superior, temporal and inferior regions measured at 1–3 mm; N6, S6, T6 and I6, the nasal, superior, temporal and inferior regions measured at 3–6 mm; HAL, spectacle lenses with highly aspherical lenslets; SAL, spectacle lenses with slightly aspherical lenslets; SVL, single vision lenses; HAL group, continued wearing HAL for 3 years; SAL-HAL group, switched from wearing SAL for 2 years to wearing HAL for 1 year; SVL-HAL group, switched from wearing SVL for 2 years to wearing HAL for 1 year

When comparing the 3-year changes in the ChT in the HAL, SAL-HAL, and SVL-HAL groups, significant differences were observed in the first 2 years, but these differences were not seen after switching to wearing the HAL (Fig. 2). In the SVL-HAL group, the ChT initially decreased while wearing the SVL but increased in the first 6 months after switching, followed by a subsequent decrease. The ChT in the SAL-HAL group increased in the first 6 months and decreased in the following year and a half when wearing SAL; upon switching to HAL, no significant changes were observed. Participants who continued wearing HAL for 3 years experienced choroidal thickening during the first year but choroidal thinning during the second year, and these changes were not significant throughout the third year.

## Discussion

During the third year, participants who consistently wore or switched to wearing HAL exhibited significantly less choroidal thinning than did children who wore SVL. Wearing HAL for 3 years resulted in choroidal thickening only during the first year, thinning in the second year and stabilization in the third year.

Several studies have demonstrated the efficacy of interventions such as orthokeratology lenses [7, 16], multifocal contact lenses [8, 17], spectacle lenses with myopic defocus signals [9, 13], atropine [18] and repeated low-level red light [19, 20] in slowing myopia progression while also leading to choroidal thickening. However, these studies reported only ChT changes for a maximum duration of 2 years, and longer-term findings are lacking. Here, participants who wore HAL for three years exhibited relatively stable ChT during the third year. Our previous study found changes in ChT for participants who wore HAL for 2 years where there was an initial increase in ChT during the first year followed by a decrease in the second year; the extent of thinning was similar to the initial thickening observed in the first year [13]. We speculated that the peak effect of the myopia control lenses on choroidal thickening occurred within the first year, and afterwards, either the lenses no longer caused choroidal thickening, or that the amount of thickening was less than the age-related thinning. This was due to the retina gradually “adapting” to the myopic control signals provided by the lenses, thereby weakening their role in myopia control and losing the continued thickening effect of the choroid. However, compared with children wearing the SVL, which exhibited significant choroidal thinning, children in the HAL group demonstrated no substantial changes in ChT, which indicated that the subtle thickening effect caused by the lenses counteracted the thinning effect resulting from increases in AL and age. This finding suggested that although the impact of lens-induced increases in ChT may diminish over time, it does not completely disappear.

The effect on choroid and myopia control was less pronounced in the SVL-HAL group in the third year than in the HAL group in the first year [14]. Notably, participants in the SVL-HAL group were older during their third year and had greater myopia and greater AL than participants in the HAL group who were during their first year. This difference implied a steeper retinal shape and potentially weakened the effect of peripheral myopic defocus from wearing HAL. However, we did not find any correlations between baseline age, refractive error, or AL and myopia progression or changes in the ChT in the HAL group. Chamberlain et al. also discovered that age and baseline myopia were found to be independent factors influencing myopia control efficacy among children who wore Misight lenses for 3 years [21]. Therefore, further verification is required regarding assumptions about age, baseline myopia, and their impact on myopia control.

The lenslets of SAL are less aspherical than those of the HAL [10], apparently resulting in a decreased thickening effect and a quicker “retinal adaptation” to myopic control signals. The SAL-HAL group exhibited an increase in the ChT only during the first half of the year when they wore SAL. Upon switching to wearing HAL, the changes in ChT in the third year in the SAL-HAL group were less pronounced than those in the SVL-HAL group but more pronounced than those in the HAL group. We speculated that when the participants switched from SAL to HAL, only the additional myopic defocus signals (the difference in signals between two lenses) had an impact. For the SVL-HAL group, all signals from the HAL had an impact; however, no new signals were induced for the HAL group. Therefore, it can be inferred that the effects of SAL-HAL lie between those of the HAL and SVL-HAL groups. Based on this conjecture, we hypothesized that augmenting defocus signals after a period of use, such as 1 year, may be more beneficial for controlling myopia progression.

When comparing changes in the AL and ChT between the HAL, SAL-HAL and SVL-HAL groups over 3 years, the changes in the AL were significantly smaller in the HAL group than in the SVL-HAL group at all visits (all *P* < 0.001) [14]. However, the changes in the ChT were no longer significantly different among the three groups at the end of the 3-year follow-up. This may be attributed to the greater variability in ChT changes among participants relative to the AL changes. Therefore, a larger change is required for significant differences to emerge. Additionally, while there was a significant correlation between the AL and ChT changes in both the HAL and SAL-HAL groups, this correlation was not observed in the SVL-HAL group. Therefore, choroidal changes cannot fully explain the myopia control effect of HAL.

There were several limitations in this study. First, we did not strictly enforce a consistent examination time for each participant across all visits, instead we allowed them to schedule between 9 a.m. and 4 p.m.; thus, we could not completely eliminate the potential impact of circadian rhythm on the ChT. In a follow-up study with a large sample size of children, it is difficult to require that participants and their parents adhere to a strict examination schedule for all visits. As this was a randomized controlled study, follow-up dates were determined by the protocol’s follow-up window, while specific times were chosen by the parents. We did not intervene in any participant or group timing choices; therefore, we believe that circadian rhythm had only a minimal effect, if any, on the intergroup comparisons. Second, OCT measurements were taken after cycloplegia, which may have affected the ChT [22]. However, as all groups at all visits experienced cycloplegia during the examinations, we assumed that its effects were negligible when comparing intra- and inter-group changes. Previous studies have shown that thickening and thinning first occur in the medium and large blood vessel layers and change the choroidal vascular index [23–26]. Specific changes in choroidal tissue may be more meaningful for myopia and myopia control. However, the thickness of different choroidal layers and the choroidal vascular index were not measured as they were outside the scope of the study.

## Conclusion

Compared with children wearing SVL, children who continued wearing or switched to wearing HAL exhibited significant inhibition of choroid thinning in the third year. Although the 3-year duration of HAL wear no longer produced a thickening effect on the choroid, it still effectively inhibited choroidal thinning. Compared to children who originally wore HAL or who had been wearing HAL, children who switched from SVL to HAL were older, more myopic and less likely to experience myopia control and choroid thickening. In the final 6 months, the HAL, SAL-HAL, and SVL-HAL groups all exhibited similar changes in ChT, suggesting that if children continued wearing HAL, comparable changes could still occur across all the three groups. However, children who wore HAL for 3 years tended to exhibit greater ChT than did those who switched from SVL or SAL to HAL. Therefore, if a thicker choroid is considered a protective factor against myopia and high myopia, we recommend early initiation of myopia control lenses for children.

## Data Availability

The datasets used and analyzed for the present study are available from the corresponding authors upon reasonable request.

## Abbreviations

HAL: Spectacle lens with highly aspherical lenslets
SVL: Single vision lens
SAL: Spectacle lens with slightly aspherical lenslets
AL: Axial length
SER: Spherical equivalent refractive error
OCT: Optical coherence tomography
RM-ANOVA: Repeated-measures analysis of variance

## References

[1] Wu H, Chen W, Zhao F, Zhou Q, Reinach PS, Deng L (2018). Scleral hypoxia is a target for myopia control. Proc Natl Acad Sci U S A.

[2] Zhou X, Zhang S, Yang F, Yang Y, Huang Q, Huang C (2021). Decreased choroidal blood perfusion induces myopia in guinea pigs. Invest Ophthalmol Vis Sci.

[3] Zhou X, Zhang S, Zhang G, Chen Y, Lei Y, Xiang J (2020). Increased choroidal blood perfusion can inhibit form deprivation myopia in guinea pigs. Invest Ophthalmol Vis Sci.

[4] Xiong S, He X, Deng J, Lv M, Jin J, Sun S (2017). Choroidal thickness in 3001 Chinese children aged 6 to 19 years using swept-source OCT. Sci Rep.

[5] Read SA, Alonso-Caneiro D, Vincent SJ, Collins MJ (2015). Longitudinal changes in choroidal thickness and eye growth in childhood. Invest Ophthalmol Vis Sci.

[6] Chiang ST, Turnbull PRK, Phillips JR (2020). Additive effect of atropine eye drops and short-term retinal defocus on choroidal thickness in children with myopia. Sci Rep.

[7] Li Z, Hu Y, Cui D, Long W, He M, Yang X (2019). Change in subfoveal choroidal thickness secondary to orthokeratology and its cessation: a predictor for the change in axial length. Acta Ophthalmol.

[8] Prieto-Garrido FL, Villa-Collar C, Hernandez-Verdejo JL, Alvarez-Peregrina C, Ruiz-Pomeda A (2022). Changes in the choroidal thickness of children wearing MiSight to control myopia. J Clin Med.

[9] Chun RKM, Zhang H, Liu Z, Tse DYY, Zhou Y, Lam CSY (2023). Defocus incorporated multiple segments (DIMS) spectacle lenses increase the choroidal thickness: a two-year randomized clinical trial. Eye Vis (Lond).

[10] Bao J, Yang A, Huang Y, Li X, Pan Y, Ding C (2022). One-year myopia control efficacy of spectacle lenses with aspherical lenslets. Br J Ophthalmol.

[11] Gantes-Nuñez J, Jaskulski M, López-Gil N, Kollbaum PS (2023). Optical characterisation of two novel myopia control spectacle lenses. Ophthalmic Physiol Opt.

[12] Bao J, Huang Y, Li X, Yang A, Zhou F, Wu J (2022). Spectacle lenses with aspherical lenslets for myopia control vs single-vision spectacle lenses: a randomized clinical trial. JAMA Ophthalmol.

[13] Huang Y, Li X, Wu J, Huo J, Zhou F, Zhang J (2023). Effect of spectacle lenses with aspherical lenslets on choroidal thickness in myopic children: a 2-year randomised clinical trial. Br J Ophthalmol.

[14] Li X, Huang Y, Yin Z, Liu C, Zhang S, Yang A (2023). Myopia control efficacy of spectacle lenses with aspherical lenslets: results of a 3-year follow-up study. Am J Ophthalmol.

[15] Hirasawa K, Shoji N, Yoshii Y, Haraguchi S (2014). Comparison of Kang’s and Littmann’s methods of correction for ocular magnification in circumpapillary retinal nerve fiber layer measurement. Invest Ophthalmol Vis Sci.

[16] Li Z, Cui D, Hu Y, Ao S, Zeng J, Yang X (2017). Choroidal thickness and axial length changes in myopic children treated with orthokeratology. Cont Lens Anterior Eye.

[17] Amorim-de-Sousa A, Pauné J, Silva-Leite S, Fernandes P, Gozález-Méijome JM, Queirós A (2023). Changes in choroidal thickness and retinal activity with a myopia control contact lens. J Clin Med.

[18] Yam JC, Jiang Y, Lee J, Li S, Zhang Y, Sun W (2022). The association of choroidal thickening by atropine with treatment effects for myopia: two-year clinical trial of the Low-concentration Atropine for Myopia Progression (LAMP) Study. Am J Ophthalmol.

[19] Liu G, Li B, Rong H, Du B, Wang B, Hu J (2022). Axial length shortening and choroid thickening in myopic adults treated with repeated low-level red light. J Clin Med.

[20] Chen H, Wang W, Liao Y, Zhou W, Li Q, Wang J (2023). Low-intensity red-light therapy in slowing myopic progression and the rebound effect after its cessation in Chinese children: a randomized controlled trial. Graefes Arch Clin Exp Ophthalmol.

[21] Chamberlain P, Peixoto-de-Matos SC, Logan NS, Ngo C, Jones D, Young G (2019). A 3-year randomized clinical trial of MiSight lenses for myopia control. Optom Vis Sci.

[22] Ye L, Li S, Shi Y, Yin Y, He J, Zhu J (2021). Comparisons of atropine versus cyclopentolate cycloplegia in myopic children. Clin Exp Ophthalmol.

[23] Li J, Zhu L, Zhu R, Lu Y, Rong X, Zhang Y (2021). Automated analysis of choroidal sublayer morphologic features in myopic children using EDI-OCT by deep learning. Transl Vis Sci Technol.

[24] Alshareef RA, Khuthaila MK, Januwada M, Goud A, Ferrara D, Chhablani J (2017). Choroidal vascular analysis in myopic eyes: evidence of foveal medium vessel layer thinning. Int J Retina Vitreous.

[25] Devarajan K, Sim R, Chua J, Wong CW, Matsumura S, Htoon HM (2020). Optical coherence tomography angiography for the assessment of choroidal vasculature in high myopia. Br J Ophthalmol.

[26] Rasheed MA, Singh SR, Invernizzi A, Cagini C, Goud A, Sahoo NK (2018). Wide-field choroidal thickness profile in healthy eyes. Sci Rep.

